# Intracranial pulsatile pressure gradient: a hypothesis and the potential role of dual intracranial pressure monitoring

**DOI:** 10.3389/fsurg.2026.1965112

**Published:** 2026-10-22

**Authors:** Georgios Tsermoulas

**Affiliations:** 1 Department of Neurosurgery, University Hospitals Birmingham NHS Foundation Trust, Birmingham, United Kingdom; 2 Metabolism and Systems Science, School of Medical Sciences, University of Birmingham, Birmingham, United Kingdom

**Keywords:** amplitude, cerebrospinal fluid, intracranial pressure, intracranial pressure monitoring, pulsatility

## Abstract

Intracranial pressure (ICP) monitoring is widely used in neurosurgical practice and the two key ICP variables, mean pressure and pulse amplitude, provide important information about intracranial compliance and cerebrospinal fluid dynamics. Mean ICP is assumed to be uniformly distributed and monitoring at a single intracranial location is considered representative of global ICP, but whether pulse amplitude is spatially uniform within the intracranial cavity remains uncertain. This article proposes the hypothesis that ICP waves undergo spatial attenuation within the skull resulting in an intracranial pulsatile pressure gradient. The hypothesis is supported by physiological principles of wave propagation and by evidence demonstrating attenuation of pressure waves along the craniospinal axis, although intracranial attenuation has not yet been experimentally demonstrated. This manuscript proposes that this hypothesis could be investigated using synchronous ICP measurements at two intracranial sites separated by a defined distance. It also describes the concept of a dual-sensor intracranial catheter, incorporating deep and superficial pressure sensors, that could provide simultaneous measurements and quantify the hypothetical spatial attenuation. Demonstrating an intracranial pulsatile pressure gradient could provide new insights into intracranial hydrodynamics and the interpretation of ICP pulsatility, with potential implications for neurosurgical conditions including traumatic brain injury, normal pressure hydrocephalus, idiopathic intracranial hypertension, and Chiari malformation.

## Introduction

Intracranial pressure (ICP) is a complex physiological parameter regulated by multiple factors, and its accurate interpretation is essential for the management of various neurosurgical conditions. Our current understanding of ICP originates from studies on continuous intraventricular pressure recording that began in the mid-20th century, which led to the identification of important ICP metrics (1). Modern ICP monitoring enables analysis of key variables, such as mean pressure, amplitude, waveform morphology, and wave patterns, whose clinical relevance varies depending on the underlying condition and therefore requires context-specific interpretation.

Invasive ICP monitoring is standard practice in the management of many neurosurgical conditions. Four main types of invasive monitoring systems are available based on the location of the pressure sensor: intraventricular, parenchymal, epidural, and subarachnoid (Figure 1). The intraventricular system, commonly referred to as an external ventricular drain, is considered the gold standard. The ventricular catheter is connected to a fluid-coupled strain-gauge transducer, and the system is calibrated to a zero-reference point at the level of the tragus, approximating the anatomical position of the foramen of Monro (2). Such techniques provide information on ICP dynamics at a single location within the intracranial cavity. This is generally not considered a limitation, as the intracranial cavity is assumed to behave as a uniform, non-compartmentalized space under physiological conditions, allowing for an even distribution of pressure. Furthermore, the contribution of hydrostatic pressure gradient within the skull is considered negligible. Therefore, recordings of mean pressure, waveform morphology, and characteristic wave patterns are regarded as representative of global ICP.

**Figure 1:**
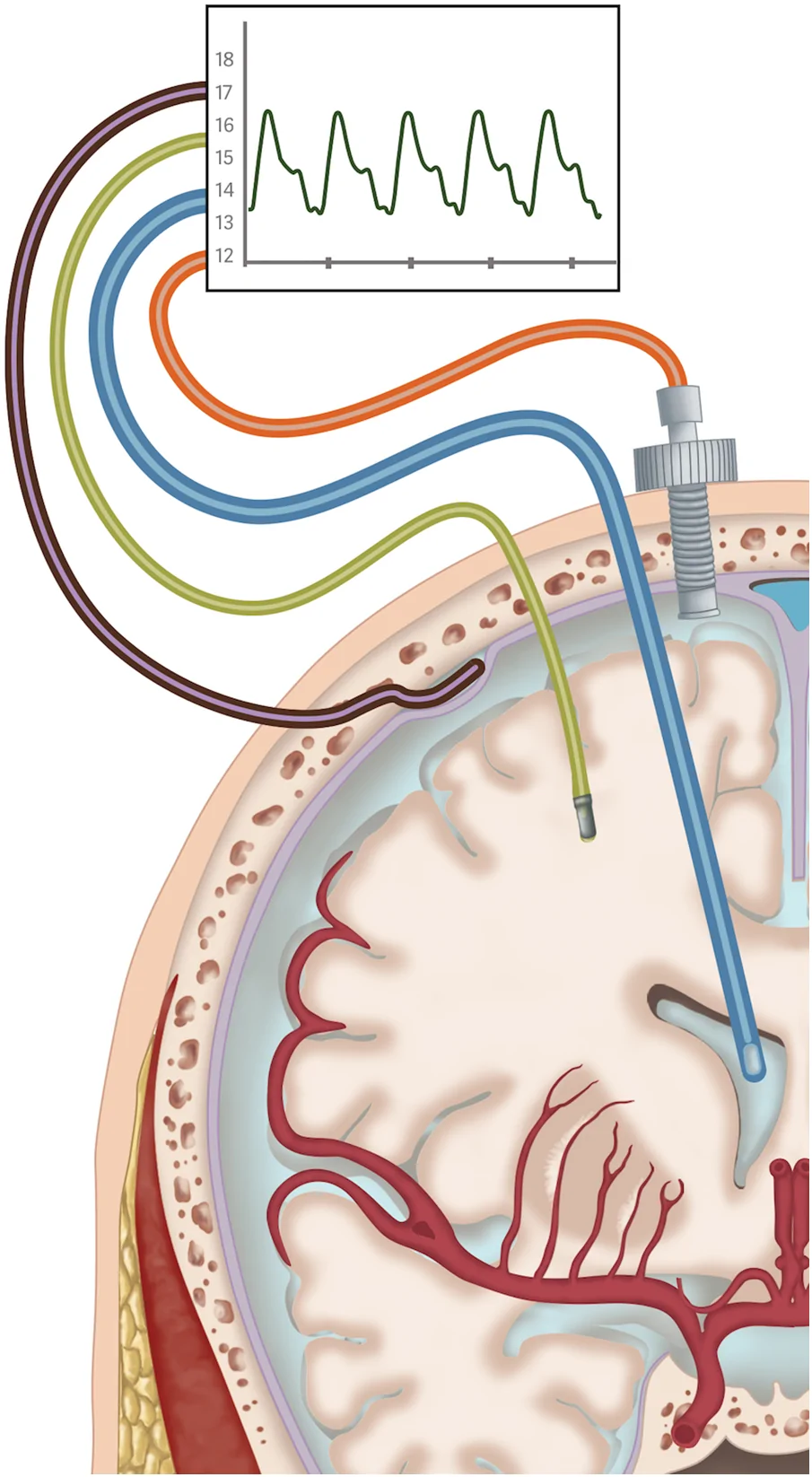
Invasive intracranial pressure (ICP) monitoring methods. Current methods use a sensor placed at one intracranial location (from right to left: epidural, parenchymal, intraventricular, subdural). The output of the system consists of an ICP curve with mean and amplitude. While mean ICP is evenly distributed throughout the intracranial cavity, these techniques cannot capture potential attenuation of pulse amplitude.

However, the assumption that ICP monitoring at a single intracranial location accurately measures pulse amplitude, should be questioned. Despite the widespread use of single-site ICP monitoring and previous studies comparing ICP measurements across different intracranial compartments, it remains unknown whether ICP pulse amplitude undergoes spatial attenuation within the cranial cavity. Amplitude represents the difference between the peak and trough ICP, and from a physiological perspective, it is plausible that ICP waves may attenuate as they propagate within the intracranial cavity. The present article proposes the hypothesis that intracranial pulse amplitude undergoes spatial attenuation and describes a testable approach using synchronous dual-site ICP monitoring. Amplitude is the second most importance metric of ICP after mean pressure (3), and addressing this question is important for advancing our understanding of intracranial hydrodynamics with potential implications for disorders of cerebrospinal fluid (CSF) circulation and conditions associated with altered ICP.

## Physiological basis of intracranial pressure pulsatility

This section explores physiological aspects of ICP that are related to hypothetical attenuation of pressure waves as they propagate within the intracranial cavity. It describes the contribution of blood flow to the generation of ICP and explains the theoretical basis for the presence of an intracranial pulsatile pressure gradient.

The ICP is the resultant pressure generated by the interaction of several constituents within the intracranial cavity, and the pulsatile blood flow is the primary generator of ICP pulse wave. With every heartbeat, approximately 20% of the cardiac stroke volume enters the basal subarachnoid space via the two carotid and two vertebral arteries, and based on the concept of the Windkessel effect, the kinetic energy of the blood flow is partly dissipated with the expansion and recoil of the elastic arterial wall and partly transmitted to the CSF, generating the ICP waves (Figure 2) (4). A key characteristic of the waves is their amplitude which is defined as the difference between their peak (systolic) and trough (diastolic) value (3).

**Figure 2:**
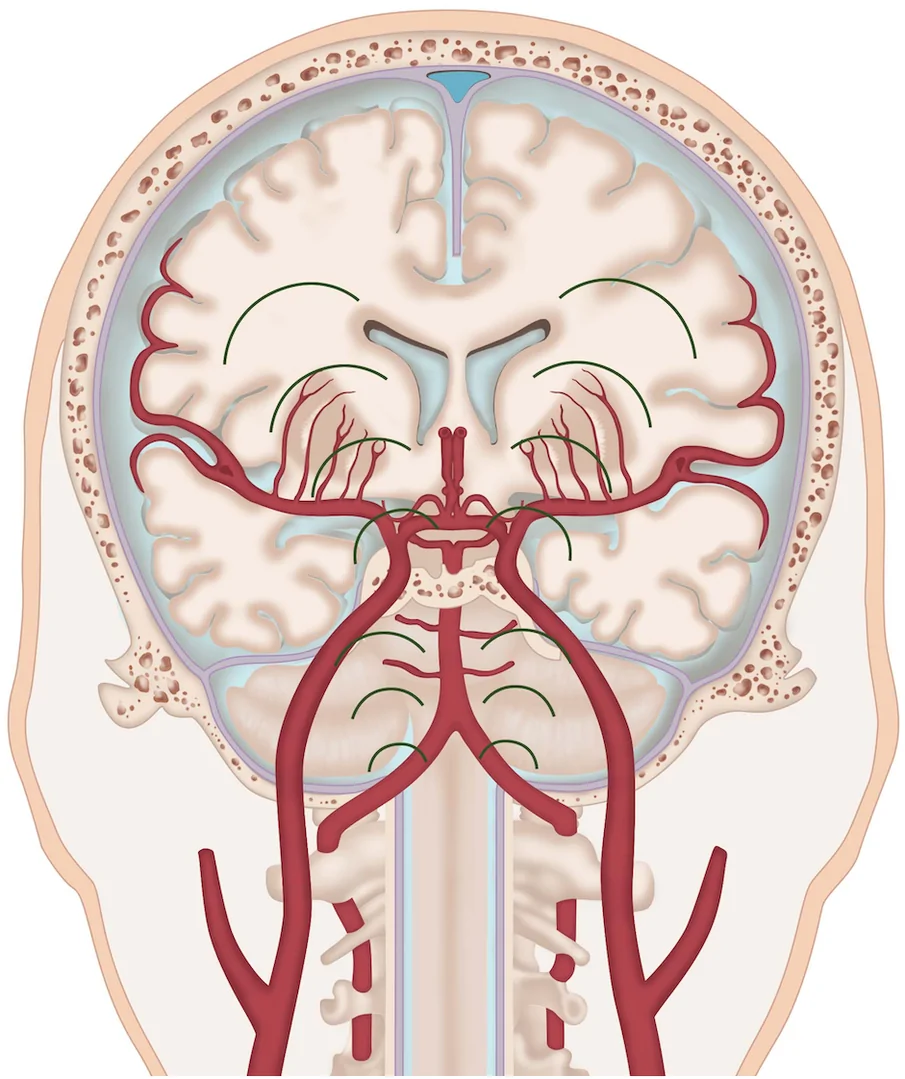
The intracranial pressure waves are primarily generated at the skull base where the two internal carotid and the two vertebral arteries enter the subarachnoid space. It is reasonable to assume that the wave height is greatest at the point of origin and gradually decreases as the waves propagate away from the source. Pulse amplitude attenuation has been experimentally confirmed along the craniospinal pathway, but the presence of an intracranial pulsatile pressure gradient remains theoretical.

According to basic wave mechanics, the energy of the ICP waves is distributed over an increasingly larger area as they spread outward from their origin at the skull base towards the rest of the intracranial cavity. In addition, portion of the waves’ energy is dissipated through mechanisms such as friction and absorption within the brain parenchyma, the main viscoelastic component of the intracranial contents. Therefore, due to both the spreading of energy and energy losses, it is logical to assume that the amplitude of waves progressively diminishes as they move farther from their source. A useful analogy is a pebble dropped into a pod where the ripples are higher at the point of impact and gradually diminish in height as the move outward. However, this assumption oversimplifies the complex fluid–solid and vascular dynamics of the cranial compartment, where wave reflection, vascular compliance, tissue properties, and compartmentalization may alter the transmission and amplitude of pressure waves. Nevertheless, the application of basic wave mechanics in this context provides a theoretical framework for investigating spatial variations in ICP wave amplitude, rather than a proof that attenuation occurs.

Attenuation of the pressure waves has been reported as they travel along the craniospinal pathway, resulting in lower amplitude at the lumbar theca compared with intracranial amplitude (5, 6). The radiculomedullary arteries entering the spinal subarachnoid space via the intervertebral foramina make only a secondary contribution to the CSF pressure waves, producing local perturbations (7). However, the spatial attenuation of ICP waves within the intracranial cavity has not been described in the literature and remains hypothetical.

## Evidence for pressure wave attenuation

Attenuation of pressure waves along the craniospinal axis has been demonstrated through simultaneous pressure measurements in the intracranial and intrathecal compartments (5, 6, 8). Whether similar wave dampening occurs within the intracranial cavity warrants investigation using an analogous approach, incorporating synchronous ICP recordings at multiple intracranial sites. Based on principles of wave physics, these recording sites should have different distance from the origin of ICP waves at the skull base.

Simultaneous ICP monitoring using two intracranial sensors has been reported in several studies, albeit addressing premises different from that of the present paper. Brean et al. (9) analyzed concurrent intraventricular and parenchymal recordings in a patient with subarachnoid hemorrhage and observed similar pulse amplitudes. Eide (10) compared parenchymal and epidural measurements in patients with idiopathic normal pressure hydrocephalus, while Eide and Sorteberg (11) performed a similar comparison in hydrocephalic patients. Both studies reported only minor differences in wave amplitude and latency and concluded that there is no significant transmantle pressure gradient to account for ventricular enlargement. In another study, Klein et al. (12) investigated the effect of CSF drainage on ICP in patients with both parenchymal and ventricular sensors and found that the amplitude values were comparable under steady-state conditions, but significantly different during CSF drainage, suggesting that ICP amplitude is potentially compartment- and measurement- dependent. Additionally, Eide et al. (13) compared ICP recordings in patients with intracranial hemorrhage using different sensor types to assess agreement between methods and found that in a subgroup of patients with two identical parenchymal sensors, differences in wave amplitude and latency were marginal.

Simultaneous supratentorial and infratentorial ICP monitoring has also been employed in posterior fossa pathologies and demonstrated higher mean infratentorial pressure (14, 15). The effect on pulse amplitude has been less studied, with a case report of a patient with cerebellar infarction showing similar pulse amplitudes between the two compartments despite a higher posterior fossa mean pressure (16). This suggested that the transtentorial pressure gradient may predominantly affect mean pressure rather than amplitude, and this finding requires confirmation in larger cohorts.

These studies examined transmantle pressure gradients, inter-sensor comparability and pressure differences across distinct intracranial compartments. However, none of them were specifically designed to investigate the existence of a pulsatile pressure gradient between the skull base and the cranial vault. Most of them used sensors from different manufacturers and placed them in intracranial compartments with different viscoelastic properties, without accounting for their relative distance from the skull base. It is unclear whether the minimal amplitude differences demonstrated is some studies represent true physiological differences or fall within the margin of error associated with the recording methodology. Therefore, definitive conclusions regarding the spatial distribution and attenuation of ICP pulsatility within the cranial cavity cannot be drawn from the available evidence.

## Hypothesis of an intracranial pulsatile pressure gradient

This paper hypothesizes that intracranial pressure pulse amplitude is not spatially uniform and progressively attenuates as pressure waves propagate away from their source near the skull base, where the main intracranial arteries enter the subarachnoid space. It further hypothesizes that this attenuation can be detected by synchronous ICP monitoring obtained at two intracranial locations with a defined distance from the skull base, with the deeper sensor expected to measure greater pulse amplitude than the more superficial one. The proposed hypothesis is testable using dual ICP monitoring and the following section describes a potential experimental approach to investigate it.

## Experimental approach: dual intracranial pressure monitoring

This paper recommends that a hypothetical intracranial pulsatile gradient should be investigated with a study that will employ synchronous ICP monitoring with the same method at two distinct intracranial sites of similar viscoelastic properties, that have a different and defined distance from the skull base. The brain parenchyma is the intracranial medium that would best control placement of sensors at different distance from the skull base and therefore two parenchymal sensors are required. Using existing technology, such approach would require dual catheterization with accurate measurement of the relative distance between the two parenchymal sensors (Figure 3). This paper does not advocate dual catheterization of parenchyma, as safer alternative approaches, including non-invasive techniques, may become available.

**Figure 3:**
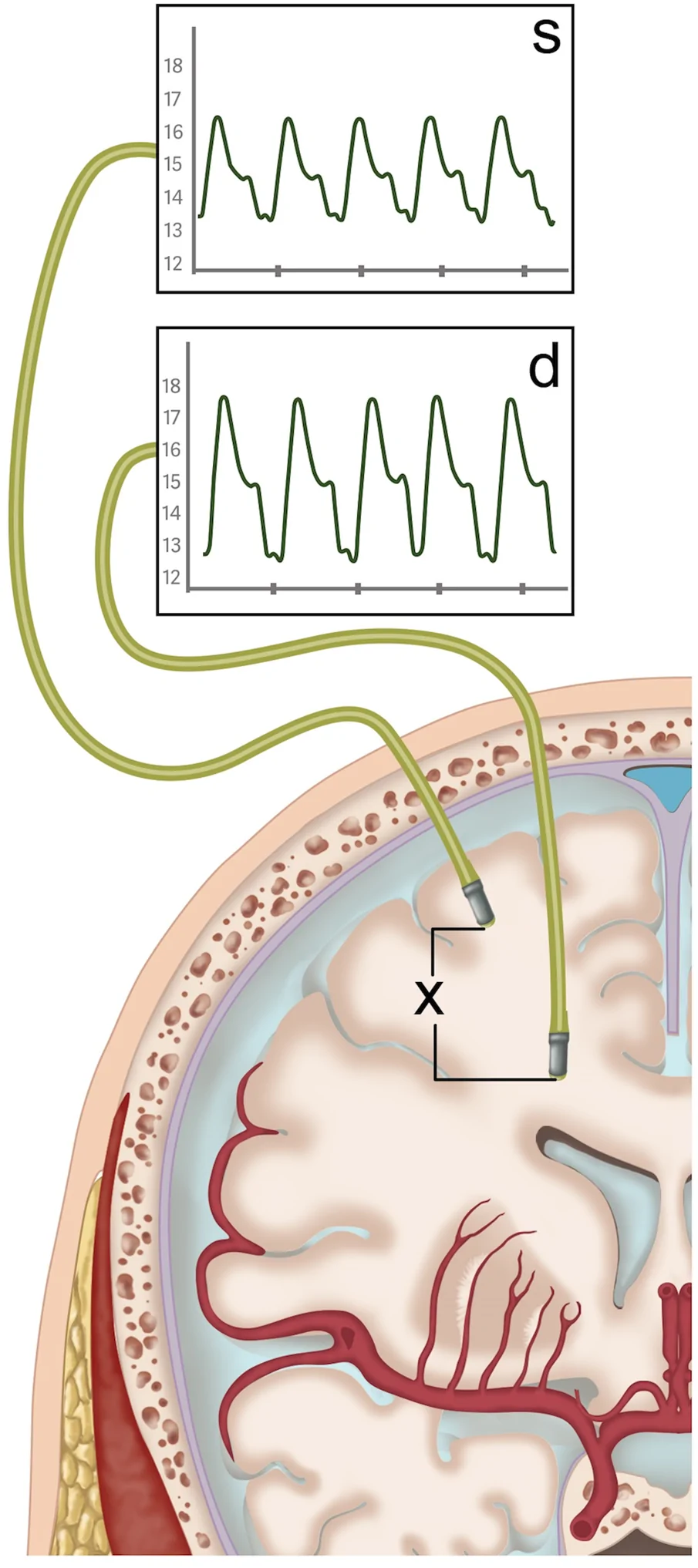
Using existing technology, a hypothetical intracranial pulsatile pressure gradient could be investigated using two parenchymal sensors positioned at different distances from the skull base (x = distance). If pressure waves attenuate within the intracranial cavity, the deeper sensor (d) would be expected to record a higher pulse amplitude compared to the more superficial sensor (s).

An alternative approach that is described here for the first time is the concept of a single system, similar to the existing invasive ICP monitors, where the intracranial catheter has an additional sensor to the one at the tip. The additional sensor would be more superficial, along the catheter at a defined distance from the tip sensor (Figure 4). Such catheter would not require the technical challenge of measuring the distance between independent sensors. Before clinical use, both sensors should undergo calibration under identical pulsatile pressure conditions to verify comparable amplitude measurements, with a negligible or absent difference indicating good inter-sensor agreement. The risk of hemorrhage of existing ICP monitoring systems is less than 1% (17), but the safety of the proposed device would need to be evaluated.

**Figure 4:**
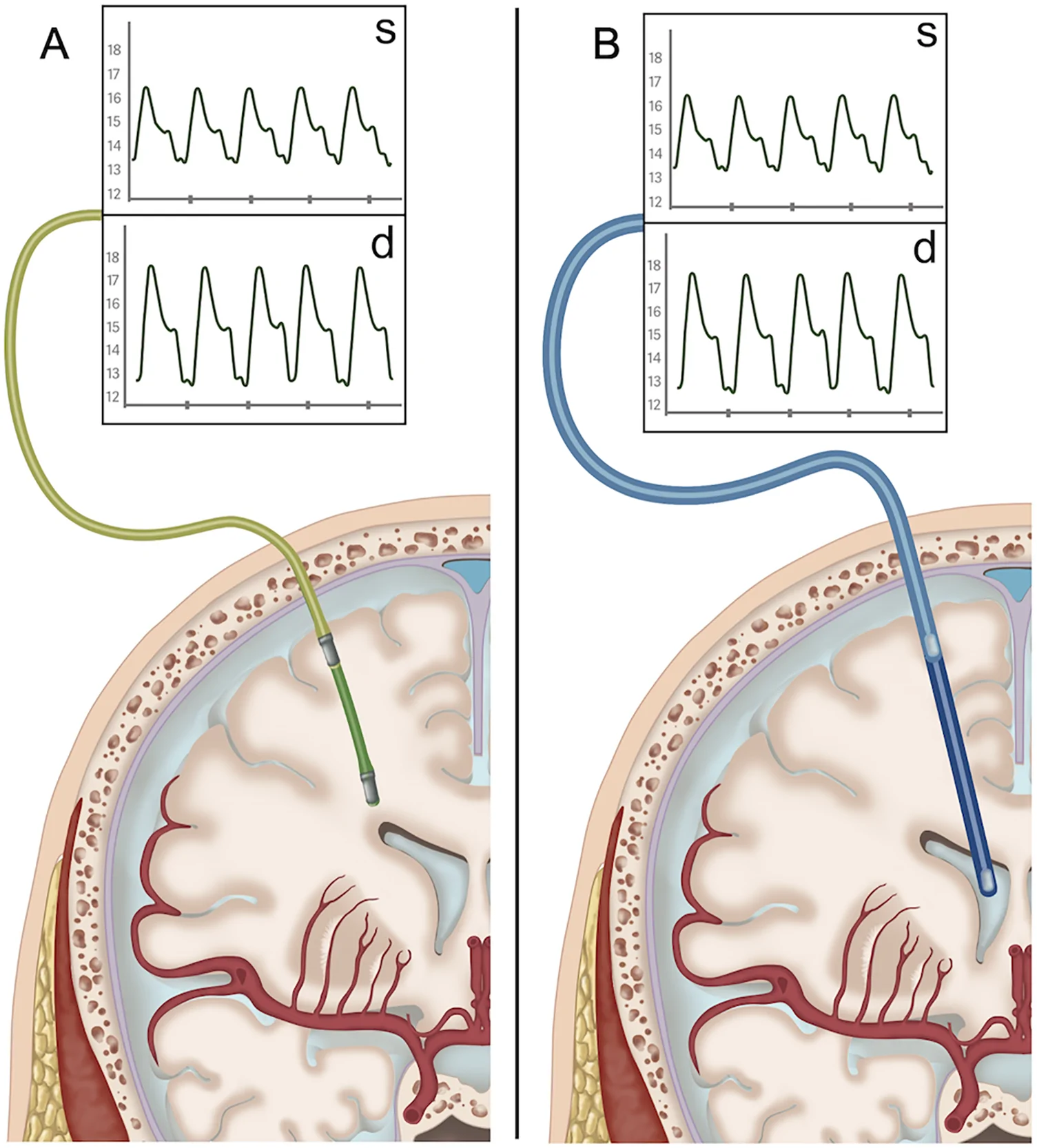
A system for dual intracranial pressure monitoring would consist of a single intracranial catheter with two sensors, a superficial (s) and a deep (d) one. The output would be two synchronous ICP recordings. The amplitude from the deep recording **(d)** is expected to be greater. The left side **(A)** depicts the dual parenchymal catheter, and the right side **(B)** depicts the combined parenchymal-intraventricular catheter.

The hypothetical intracranial pulsatile gradient might be smaller than the craniospinal gradient due to the small size of the intracranial cavity compared to the length of the spine. Accordingly, the shorter the distance between the recording sensors, the smaller the absolute amplitude difference and the harder it would be to detect differences in the amplitude of the pressure waves. On the other hand, the longer the distance between the sensors, the deeper the catheter will need to be inserted, increasing the surgical risk to the critical structures deep in the brain. The likelihood is that the intracranial pressure gradient is subtle, all confounding factors should be controlled in order to increase accuracy, and the considerations about the length of the intracranial catheter should include the diagnostic yield versus the surgical risk.

### Potential dual-sensor configurations

The four intracranial compartments currently used to measure ICP are the convexity epidural and subdural spaces, the lateral ventricle and the brain parenchyma. A dual ICP monitoring system would use a single intracranial catheter with two sensors placed either within the same or different compartments. Several potential combinations are possible and the three that may be most appropriate and technically feasible are described below.

#### Dual parenchymal ICP monitor

This system has a deep sensor at the tip of an intracranial wire, like the current parenchymal sensors, and an additional proximal sensor (Figure 4A). Insertion follows the standard technique with both sensors placed within the parenchyma of the frontal lobe and the catheter orientated as perpendicular as possible to the surface area that the carotid and vertebral arteries project onto at the skull base. Existing ICP systems allow an implantation depth of 2–4 cm (18), and therefore the distance between the sensors should be towards the longer end of the range to increase the chance of detecting subtle difference. However, the proposed placement must remain within the white matter and avoid deeper structures, in order to minimize the risk of neurological and vascular injury. The advantage of this system is that it employs the same sensors within the same medium.

#### Combined parenchymal—intraventricular ICP monitor

This system is similar to an intraventricular catheter and incorporates a superficial parenchymal sensor (Figure 4B). However, conventional ICP monitoring with an external ventricular drain is unsuitable here, because a transducer connected to a 3-way stopcock outside the head would record a significantly attenuated amplitude. Therefore, the deep sensor should be placed at the catheter tip within the lateral ventricle. Compared with the dual parenchymal system, the main advantage is the greater inter-sensor distance, as the distance from the cortex to the foramen of Monro is approximately 6 cm. The disadvantage is that the sensors are placed in media with different viscoelastic properties.

#### Combined subarachnoid—intraventricular ICP monitor

This system incorporates a subarachnoid and an intraventricular sensor. Its advantages are that both sensors are placed within CSF and their distance is slightly longer compared to the combined parenchymal—intraventricular one. However, it may be technically more challenging to insert it compared with the other two catheters.

### Quantification of intracranial pressure amplitude attenuation

Based on the hypothetical attenuation of the ICP waves, the expectation is that the amplitude of the deep sensor (AMPd) will be greater compared to the amplitude of the superficial sensor (AMPs) and the intracranial pulsatile gradient (IPG) is calculated based on the formula IPG=(AMPd—AMPs)/x where x is the inter-sensor distance. This measures the absolute spatial attenuation of the amplitude, with units of amplitude per unit distance.

The above metric is influenced by the overall magnitude of the amplitude, whereas a relative amplitude change is more meaningful because it reduces dependence on baseline pulsatility and allows comparison between different pathologies and patients. The formula for the “normalized” gradient that quantifies the drop relative to the overall pulsatility is IPGnorm=(AMPd—AMPs)/[(AMPd+AMPs)/2 × x]. The amplitude units cancel during normalization, and the result is expressed as relative attenuation per unit distance. The resulting value can be multiplied by 100 to express the relative attenuation as a percentage.

## Discussion

This paper proposes that there is a theoretical basis for the attenuation of ICP waves, based on physiologically plausible assumptions and extrapolation from evidence reported in the literature. It describes how dual ICP monitoring would measure the potential intracranial pulsatile pressure gradient to test the above hypothesis. If confirmed, it will enhance our understanding of intracranial hydrodynamics and the pathophysiology of diseases associated with altered ICP pulsatility.

Pulse amplitude is a key variable of ICP and a measure of intracranial compliance. Abnormal amplitude has been linked to several neurosurgical conditions including traumatic brain injury, normal pressure hydrocephalus, idiopathic intracranial hypertension and Chiari malformation type 1 (3). Increased ICP pulsatility has also been linked to neurodegenerative conditions and the terms “pulsatile” and “pulse-wave” encephalopathy have been proposed to describe the harmful impact of increased amplitude on the brain (19–21).

The contribution of ICP pulsatility to idiopathic normal pressure hydrocephalus is of particular interest, because some studies have identified elevated amplitude as a characteristic feature of the condition (22, 23), while others have not demonstrated a significant increase (24, 25). If an intracranial pulsatile gradient exists, it could explain these discrepancies, as measurements obtained at a single location may fail to detect pathological pulsatility. Wave attenuation may “normalize” the amplitude at the periphery where it is usually recorded and this is consistent with the hypothesis that compensatory structural changes occur in normal pressure hydrocephalus to maintain ICP homeostasis (26).

### Limitations

The manuscript does not provide experimental evidence for an intracranial pulsatile pressure gradient but proposes a hypothesis and a methodology by which the hypothesis could be tested. Amplitude attenuation within the intracranial compartment is likely to be subtle, and dual ICP monitoring may therefore lack sufficient diagnostic yield to detect it. A difference of approximately 2 mmHg has been reported between the intracranial and intrathecal recordings (5, 8), and therefore the difference within the skull may be a fraction of that difference. Studies that failed to detect a transmantle pressure gradient also suggested that the current invasive monitoring techniques may lack the precision required to detect small pressure differences, prompting the proposal of more refined computational modelling approaches (27). The longer the inter-sensor distance the higher the chance of detecting a small difference, but this has to be weighed against the risk to eloquent brain and vascular structures near the skull base. Finally, the hypothesis described in this paper does not account for the different viscoelastic properties of the various intracranial components, the compartmentalized nature of intracranial CSF, or the compartmentalization of the intracranial cavity that may occur under certain pathological conditions. Nonetheless, this assumption is based on a physiologically plausible hypothesis and exploring the presence of a hypothetical intracranial pulsatile gradient is an important research question.

In summary, intracranial pulse amplitude may not be uniform and may attenuate as ICP waves propagate away from their site of origin near the skull base, where the major intracranial arteries enter the basal subarachnoid space. The existence of a hypothetical intracranial pulsatile gradient warrants investigation and this paper describes the concept of dual ICP monitoring to test this hypothesis. Exploring the presence of such a gradient would enhance our understanding of intracranial hydrodynamics and the mechanisms governing ICP pulsatility.

## Data Availability

The original contributions presented in the study are included in the article/Supplementary Material, further inquiries can be directed to the corresponding author.
